# The Influence of Thermocycling and Ultraviolet Aging on Surface Characteristics and the Repair Bond Strength of CAD/CAM Resin Nanoceramics

**DOI:** 10.3390/jfb16050156

**Published:** 2025-04-28

**Authors:** Beyza Unalan Degirmenci, Alperen Degirmenci, Zelal Seyfioglu Polat

**Affiliations:** 1 Department of Prosthodontics, University of Van Yuzuncu Yil, Van 65080, Turkey; 2 Department of Restorative Dentistry, University of Van Yuzuncu Yil, Van 65080, Turkey; adegirmenci@yyu.edu.tr; 3 Department of Prosthodontics, University of Dicle, Diyarbakır 21300, Turkey; zelalpolat@hotmail.com

**Keywords:** CAD/CAM resin nanoceramic, UV aging, surface properties

## Abstract

Background: The durability of computer-aided design/computer-aided manufacturing (CAD/CAM) resin nanoceramics in the oral environment is influenced by aging factors such as thermocycling and ultraviolet (UV) exposure. This study investigates the impact of these aging processes on surface characteristics and repair bond strength. Methods: CAD/CAM resin nanoceramic samples were divided into the following five groups: control (non-aged), 1-year and 5-year thermocycling, and 1-year and 5-year UV aging (*n* = 12). For the thermocycling procedure, the parameters employed were a temperature range of 5–55 °C with dwell times of 20 s per bath and 10,000 and 50,000 cycles; for the ultraviolet aging process, the parameters were established at a wavelength of 340 nm, an intensity of 0.55 W/m², and durations of 300 h and 1500 h. Surface roughness, microhardness, and repair bond strength were analyzed through profilometry, Vickers microhardness testing, and shear bond strength assessment, respectively. SEM, AFM, and XRD analyses were performed for structural evaluation. Results: Both thermocycling and UV aging significantly increased surface roughness (*p* < 0.001) while reducing microhardness and repair bond strength (*p* < 0.001). UV aging had a more pronounced effect, particularly after five years, leading to the highest surface roughness (Ra: 61.77 μm; Rz: 271.57 μm) and lowest microhardness properties (63.13). EDAX analysis indicated matrix degradation and an increase in inorganic filler exposure. Conclusions: Aging significantly affects the surface characteristics of CAD/CAM resin nanoceramics, with UV aging exhibiting the most detrimental impact. These findings highlight the necessity of considering long-term material stability in dentistry.

## 1. Introduction

Recent advancements in computer-aided design and computer-aided manufacturing (CAD/CAM) systems have facilitated the creation of aesthetic CAD/CAM restorations that demonstrate improved clinical outcomes [1]. Moreover, the incorporation of intraoral cameras, advanced design software, enhanced biomaterials, and digital modeling within these systems has substantially influenced the field of prosthetic and restorative dentistry [2]. This integration has contributed to the growing prevalence and acceptance of CAD/CAM systems in contemporary dental practices [3]. In response to patient preferences and the requirements of dental professionals, ceramics have emerged as the preferred materials for aesthetic restorations, applicable in both traditional production techniques and fully digital workflows [4]. The aesthetic qualities, biocompatibility, durability, and resistance to discoloration inherent in ceramic materials are the primary factors underpinning this selection [5]. However, Kassem et al. have identified several significant disadvantages associated with ceramics. These include their propensity to cause considerable wear on opposing dentition, the increased visibility of defects on the intaglio surfaces due to their brittle characteristics, and their tendency to fracture [6]. In response to prevailing challenges in the field, the market has recently introduced new CAD/CAM materials known as hybrid-resin ceramics. These innovative blocks are designed to integrate the benefits of both resin and ceramic restoration materials, thereby enhancing the performance and effectiveness of dental restorations [7].

Hybrid-resin ceramics represent advanced materials characterized by a nanohybrid structure that incorporates inorganic ceramic fillers, which have been developed utilizing cutting-edge nanotechnology [8]. The materials incorporate micro, hybrid, and nano fillers in addition to a resin matrix [9]. The resin matrix structure is composed of various dimethacrylate thermosets, including ethoxylated bisphenol-A dimethacrylate (BisEMA), ethylene glycol dimethacrylate (EGDMA), bisphenol-A-diglycidyl methacrylate (BisGMA), and urethane dimethacrylate (UDMA). The filler component consists of silica fillers in nanoscale sizes, nanocluster particles derived from these fillers, as well as zirconia and barium glass fillers [10]. Cerasmart (GC), commonly referred to as resin nanoceramics, is one of the most prominent hybrid-resin ceramic materials available. This innovative material comprises 71% silica along with barium glass nanoparticles, showcasing its advanced compositional structure [11]. This material presents several significant advantages for clinicians, including low abrasion and superior flexural properties, as well as enhanced resistance to chipping and cracking during the milling process. Moreover, it allows for accelerated milling procedures and reduces wear on milling instruments due to its low microhardness. Its structure is less brittle compared to glass ceramics, and it does not require a post-production firing process. Additionally, the material is equipped with a dedicated staining kit that satisfies high aesthetic standards, and it facilitates the efficient finishing of restorations through polishing [12,13,14]. Nevertheless, it is well recognized that CAD/CAM materials containing a resin matrix are considerably influenced by the moist environment found within the oral cavity [15]. In addition to intraoral conditions, insufficient interconnection, improper occlusal adjustments, or the presence of parafunctional habits in the patient may render the fracture of resin nanoceramic restorations inevitable [5]. Lohbauer et al. highlighted this concern in their research, noting that the initially assessed mechanical properties may not serve as a reliable criterion for evaluating the clinical performance of these materials [16].

Aging methodologies can be utilized to assess the mechanical resistance and physical properties of dental materials within the oral environment in a timely manner under standardized laboratory conditions. This approach effectively simulates long-term clinical scenarios, enabling a more accurate evaluation of material performance [17,18]. The predominant aging method favored in contemporary literature remains thermocycling [19]; however, various methods exist for treatment and degradation, including soaking in water, boiling in water, exposure to acid, autoclave processing, immersion in a water–ethanol mixture, and ultraviolet (UV) aging [20]. In the seminal research conducted by Heydecke et al., it was underscored that the incorporation of aging procedures involving light exposure and variations in moisture—referred to as the weathering process—are critical factors for the accurate assessment of the performance of non-metallic materials [21]. In the current UV aging methodology, samples are subjected to continuous variations in temperature, heat, and humidity while being exposed to visible light that emits UV radiation. This approach accounts for these additional parameters in the assessment process [1]. It is anticipated that the aging process will induce intrinsic physical or chemical modifications in materials [22]. However, it is imperative to recognize that contemporary materials may demonstrate varying degrees of resistance to mechanical and chemical degradation. Such variability is attributable to differences in filler composition and the chemistry governing the interaction between the matrix and the filler [23]. The biomechanical behavior characteristics of resin nanoceramic materials have not been extensively studied to date. The limited research findings that do exist exhibit considerable variability, which is believed to be primarily attributed to the differing aging methodologies employed in these studies [19,24]. Moreover, the absence of long-term clinical data from this perspective may lead to confusion among clinicians concerning the use of new materials. The mechanical and chemical resistance of CAD/CAM resin nanoceramics plays a crucial role in their long-term efficacy within the oral environment, where they encounter thermal fluctuations, moisture, and ultraviolet exposure. It is imperative to comprehend the degradation mechanisms of these materials over time to accurately predict their clinical durability and reduce the incidence of restoration failures. Therefore, it is crucial to conduct surface analyses, including assessments of microhardness, roughness, elemental changes, and phase alterations that occur in resin nanoceramic materials following various aging methods. Literature plays a crucial role in the selection of durable CAD/CAM materials by elucidating the impact of aging on surface properties and the strength of repair bonds. Materials exhibiting enhanced resistance to ultraviolet aging or thermal cycling are generally preferred for long-term restorations in high-stress environments. Conversely, certain materials may be more suitable for short-term applications. This knowledge aids clinicians in optimizing treatment outcomes while minimizing the frequency of repairs or replacements.

The objective of this study is to assess the impacts of thermocycling and UV aging at various cycles on the microhardness, surface roughness, and repair bond strength of CAD/CAM resin nanoceramic, in comparison to resin nanoceramic that has not undergone aging. The null hypothesis posits that there will be no significant differences in the microhardness, surface roughness, and repair bond strength values of CAD/CAM resin nanoceramics based on the aging method or cycle duration.

## 2. Materials and Methods

**Figure 1:**
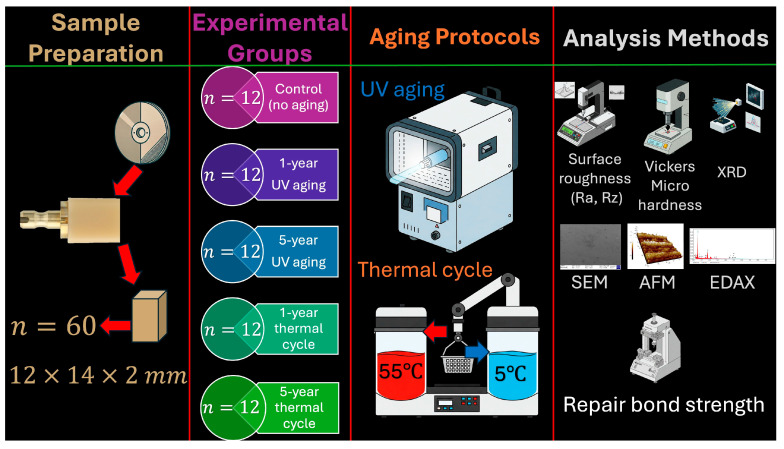
Flowchart of the study.

The experimental procedure employed in the current study is illustrated schematically in Figure 1, and the characteristics of the materials utilized are detailed in Table 1.

### 2.1. Calculation of Sample Size

The sample size for the study was determined using G*Power 3.1 software and was based on data from a previous research study [17]. The analysis, which was conducted using a normal distribution, utilized a significance level of 0.05, an effect size of 0.5, and a power of 0.95. As a result, it was determined that there would be 12 samples in each subgroup, leading to a total sample size of 60.

### 2.2. Specimen Preparation

Sample preparation was conducted in a climate-controlled laboratory maintained at a temperature of 23 ± 1 °C and a relative humidity of 50 ± 5% to mitigate environmental variability. Cerasmart block, which is a CAD/CAM resin nanoceramic, was utilized for the study. The blocks were sectioned using a diamond blade attached to a low-speed cutting device (Isomet 5000; Buehler, Lake Bluff, IL, USA) while maintaining continuous water cooling throughout the process. A total of 60 rectangular samples, each with dimensions of 12 × 14 × 2 mm^3^, were fabricated. The dimensions of all samples were measured with precision using a digital caliper (Absolute Digimatic, Mitutoyo, Kawasaki, Japan), which boasts an accuracy of 0.01 mm. Calibration of the caliper was conducted after every five samples to ensure the integrity of the measurements. Samples that did not conform to the specified size criteria were excluded from the study. The qualified samples were subsequently positioned in a 20 × 20 mm^2^ Teflon mold, which was prepared using a self-polymerizing acrylic resin (SC Acrylic Resin, Imicryl Dental, Konya, Turkey), ensuring that one surface remained fully exposed. To standardize the surfaces and eliminate any potential external scratches or defects, each sample was polished for 30 s with silicon carbide paper of 600, 800, 1000, and 1200 grit, utilizing finger pressure throughout the process. The polished samples were subjected to cleaning in an ultrasonic bath filled with distilled water to ensure the removal of any residual surface contaminants. Subsequently, they were gently dried using a clean paper towel. The dried samples underwent a thorough re-evaluation by two independent researchers, with any samples exhibiting defects being excluded from the study.

The prepared samples were categorized into five subgroups utilizing random numbers generated by a computer program, in accordance with the aging procedure and cycle duration to be implemented (*n* = 12).

### 2.3. Aging Procedure and Cycle

In the current research, two distinct aging procedures were implemented, accompanied by two varying cycle durations.

1. The samples designated as the control group were not subjected to any aging procedures. They were maintained in a container containing artificial saliva and incubated at 37 °C until the time of measurement (*n* = 12).
2. The samples that were subjected to thermocycling were placed in a thermocycling device (MOD Dental, Ankara, Turkey) containing baths with temperature ranges from 5 °C to 55 °C. The duration of each bath exposure was established at 20 s, while the transfer time between baths was fixed at 2 s [25]. The samples designated for the 1-year aging cycle underwent a testing regimen of 10,000 cycles, whereas the samples allocated for the 5-year aging cycle were subjected to 50,000 cycles, utilizing the same testing parameters.
3. A weathering machine (Ci35 Weather-Ometer; Atlas Electronic Devices Co., Illinois, USA) was used for the UV aging procedure. The specimens were subjected to a controlled exposure using a xenon arc lamp, filtered through borate borosilicate glass, at an intensity of 0.55 W/m^2^/nm, specifically measured at a wavelength of 340 nm. Aging was carried out based on the parameters established by Silva et al. [26] to replicate one year of clinical use. The conditions included a dry bulb temperature of 47 °C for light exposure and 38 °C for dark conditions, humidity levels of 50% in light and 95% in dark, a black panel temperature of 70 °C for light and 38 °C for dark, and a water temperature maintained at 50 °C. Each test cycle was designed to consist of a standardized duration of 40 min of light exposure, followed by 20 min of combined light exposure and front water spray. This was succeeded by an additional 60 min of light exposure and concluded with 60 min of darkness, accompanied by backwater spray. Due to these cycles, samples that received a total exposure energy of 150 kJ/m^2^ and a cumulative exposure time of 300 h were regarded as having aged for one year. In contrast, the exposure time was established at 1500 h for the five-year aging process. Throughout the aging processes, careful attention was devoted to monitoring the environmental conditions utilizing calibrated sensors.

### 2.4. Surface Roughness Measurement

The average surface roughness (Ra) and the difference between the highest peak and the deepest valley on each specimen’s surface (Rz) were assessed following the aging procedures utilizing a two-dimensional profilometer (Surftest 402, Mitutoyo, Tokyo, Japan). This measurement was conducted with a 5-μm diamond stylus positioned at an angle of 90 degrees. Three measurements were conducted for each specimen by rotating the specimen by 60 degrees. The tracing length was set at 3 mm, the cutoff length (λC) was specified as 0.25 mm, and the stylus speed was maintained at 0.25 mm/s. The average roughness value for each specimen was calculated and documented in micrometers. The calibration of the profilometer was periodically verified following the assessment of three specimens using a standard calibration apparatus.

### 2.5. Surface Microhardness Measurement

Following the aging procedures, the hardness values of the samples were assessed using a Vickers Microhardness Tester (HMV-2T; Shimadzu, Kyoto, Japan). Each sample surface underwent three separate indentations, applied with a load of 9.807 N and a dwell time of 20 s, ensuring that the measurement areas were closely localized. The readings were obtained under ×40 magnification. The average of the three measurements was then calculated and recorded for each respective sample. Furthermore, the calibration of the microhardness tester was rechecked after performing nine measurements on each of the three samples using a standard pretested apparatus.

### 2.6. X-Ray Diffraction (XRD) Analysis

The effects of aging conditions and applied cycle time on the crystal phases of the samples were assessed using X-ray diffraction (XRD) analysis. For this analysis, an additional sample was prepared for each subgroup, and prior to aging, the samples were polished with 15-µm diamond lapping film to achieve a mirror-like surface, which is crucial for accurate measurement. The surfaces of the aged samples were then scanned for 3 s in continuous mode using a Cu Kα tube X-ray diffractometer (Rigaku Ultima IV Multifunctional, Tokyo, Japan). The scan range was set from 5 to 90° (2θ) with X-ray current and excitation voltage maintained at 40 kV and 30 mA, respectively, a step width of 0.01°, and a scanning rate of 1.0°/min (2θ/seg).

### 2.7. Scanning Electron Microscopy (SEM), Atomic Force Microscopy (AFM), and Chemical Elemental Analysis

Following the evaluation of surface properties, three samples were randomly selected from each subgroup for analysis utilizing scanning electron microscopy (SEM) and atomic force microscopy (AFM). AFM images were acquired in contact mode, generating three-dimensional (3D) representations with a resolution of 512 × 512 pixels. These images were produced by scanning areas measuring 20 × 20 μm at a scanning speed of 1 Hz, employing an atomic force microscope (XE-100 E, Park Systems, Suwon, Republic of Korea). Nanosensors PPP-CONTSCR 10M (Park Systems), with a nominal force constant of 0.2 N/m and a resonance frequency of 23 kHz, were employed to image the specimens. The cantilever measured 225 μm in length, 1 μm in thickness, and had an average width of 48 μm. A reflective aluminum coating was applied to the backside to facilitate operation in contact mode. The atomic force microscope underwent calibration through the acquisition of measurements in a three-dimensional view of a reference sample, utilizing software calibration that involved imaging a standard sample. For the purpose of analyzing surface topography based on AFM images, the XEP Data Analysis Program, version 1.8.0, Build 48 (Park Systems), was employed. Subsequent to the completion of AFM analysis, SEM was conducted on the samples. Given the non-conductive nature of the samples, their surfaces were coated with a layer of gold and palladium using a Polaron Sputter Coater (model number SC7620, VG Microtech, West Sussex, England) for a duration of 60 s. Following this process, the samples were placed within the SEM apparatus (Gemini model SEM500, Carl Zeiss, Oberkochen, Germany), where SEM images were captured of the most representative areas at an original magnification of ×1500 and ×500. The analysis was further enhanced through the use of Energy Dispersive X-ray Spectroscopy (EDS), utilizing the Noran Voyager III system (document number M3100, Noran Instruments, Middleton, WI, USA), which was integrated into the SEM equipment.

### 2.8. Repair Bond Strength (SBS) Test

Following the measurement and analysis of the surface characteristics of the aged samples, the repair procedure commenced. A 2 mm thick green belt diamond fissure bur (837L-014 GL, Bosphorus, Istanbul, Turkey) was employed in accordance with the manufacturer’s recommendations. The grinding process was conducted under water cooling at a speed of 40,000 rpm. Throughout the procedure, the bur was maintained parallel to the sample surface. It was applied to the sample surface five times with minimal pressure, and the bur was replaced after every five samples to ensure optimal performance. Upon the completion of the bur-grinding process, the samples were subjected to a cleaning procedure lasting five minutes within an ultrasonic bath. This step was followed by a careful drying process utilizing a paper towel. Ceramic Primer II was applied in a uniform, thin layer to the surface of the sample and subsequently dried using an air spray technique. The application of a universal adhesive, specifically G-Premio Bond, was performed on the surface utilizing an applicator. The adhesive was permitted to set for 10 s and was subsequently dried using maximum air pressure for an additional 5 s. Following this process, the samples were subjected to visible blue light exposure with a wavelength range from 430 to 480 nm and a light intensity of 1400 mW/cm^2^. This was achieved utilizing an LED light device (D-Light Pro, GC Corporation, Tokyo, Japan), positioned 10 mm from the samples for a duration of 10 s. To ensure the accuracy of the LED light device’s intensity, calibration was performed by assessing one out of every five samples, with the assistance of a radiometer (LED Radiometer, SDI Dental Limited, Melbourne, Australia). A flowable composite (G-aenial Universal Flo, GC Corporation, Tokyo, Japan) utilizing the same flexible nanoceramic technology as Cerasmart was selected as the repair material. The composite was applied using a 2 mm thick mold placed over the sample surface and was cured according to the standards outlined in ISO 4049 [27]. The repaired samples were stored in distilled water at 37 °C for a duration of 24 h. The shear bond strength (SBS) test was performed using a universal testing machine (Trapezium X, Shimadzu Corp., Tokyo, Japan) with a customized steel jig. The jig featured a 2 mm diameter chisel-shaped loading head positioned 0.5 mm from the composite CAD/CAM resin nanoceramic interface to ensure uniform shear force distribution. Samples were secured in a rigid metal holder with cyanoacrylate adhesive to prevent movement during testing. Shear force at a cross-head speed of 0.5 mm/min until fracture occurred. The resulting data were recorded in megapascals (MPa). Following fracture, the samples were examined under a light microscope (TM-505, Mitutoyo, Tokyo, Japan), and the fracture patterns were categorized as adhesive, cohesive, or mixed.

### 2.9. Statistical Analysis

Data analysis was conducted using IBM SPSS V23. The Shapiro–Wilk test was employed to assess the appropriateness of the normal distribution assumption. The effects of the aging procedure and cycle time on microhardness, repair bond strength, and surface roughness values were evaluated using a two-way MANOVA. For data that did not conform to a normal distribution across three or more groups, the Kruskal–Wallis test was applied. One-way analysis of variance (ANOVA) was utilized for normally distributed data across three or more groups, with multiple comparisons carried out using Tukey’s HSD and Tamhane’s T2 tests. The Chi-square test was implemented to compare failure types between groups, and Spearman’s rho correlation coefficient was used to explore the relationship between quantitative parameters that were not normally distributed. The analysis results were presented as the mean ± standard deviation for quantitative data and as frequency (percentage) for categorical data. A significance level of *p* < 0.050 was established for the evaluation of statistical significance.

## 3. Results

Ra, Rz, microhardness, and SBS values for the samples from both the aged and non-aged (control) groups are summarized as means, standard deviations (SDs), and medians in Table 2.

**Figure 2:**
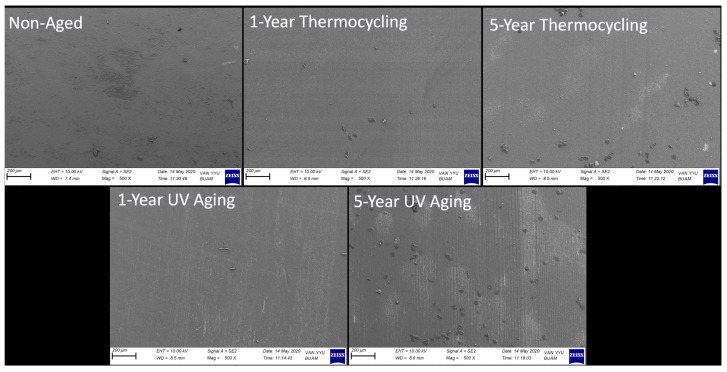
SEM image (500×) of the surface of the CAD/CAM material after aging procedures.

**Figure 3:**
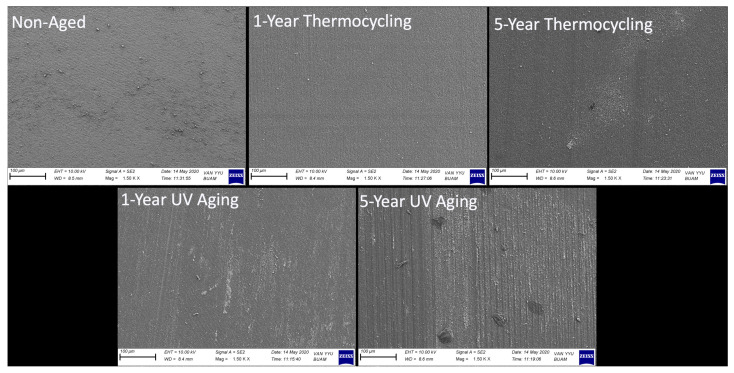
SEM image (1500×) of the surface of the CAD/CAM material after aging procedures.

The results from the two-way MANOVA indicated that the aging procedure, cycle duration, and interaction between these two factors had statistically significant effects on the Ra and Rz values of the samples (*p* < 0.001, F = 27,665.9; *p* < 0.001, F = 16,398.5; *p* < 0.001, F = 12,616.8, respectively). The highest Ra value recorded among the groups was 61.77 μm, associated with samples that underwent a 5-year UV aging cycle. This was followed by samples aged for 1 year under UV aging (40.79 μm), 5-year thermocycling (37.45 μm), 1-year thermocycling (36.08 μm), and samples from the control group (0.05 μm), respectively. The trend observed in the Ra values of the samples was similarly noted in the Rz values, with the most pronounced roughness structure measured at 271.57 μm in samples subjected to a 5-year UV aging cycle. As anticipated, the control group exhibited the lowest Rz value at just 0.22 μm. SEM microphotographs taken post-aging procedures, which support the surface roughness measurements, are presented in Figure 2 and Figure 3.

As observed in both Figure 2 and Figure 3 at higher magnification, the surfaces of the non-aged control group samples exhibit a relatively uniform structure, though not entirely free of minor irregularities. In contrast, a damage and wear pattern linked to the effects of the procedure was observed on the surfaces of samples subjected to both thermocycling and UV aging. It can be concluded that as the cycle time of the procedure increased, the wear patterns resulting from the damage became more pronounced, with a corresponding increase in crevice depths.

**Figure 4:**
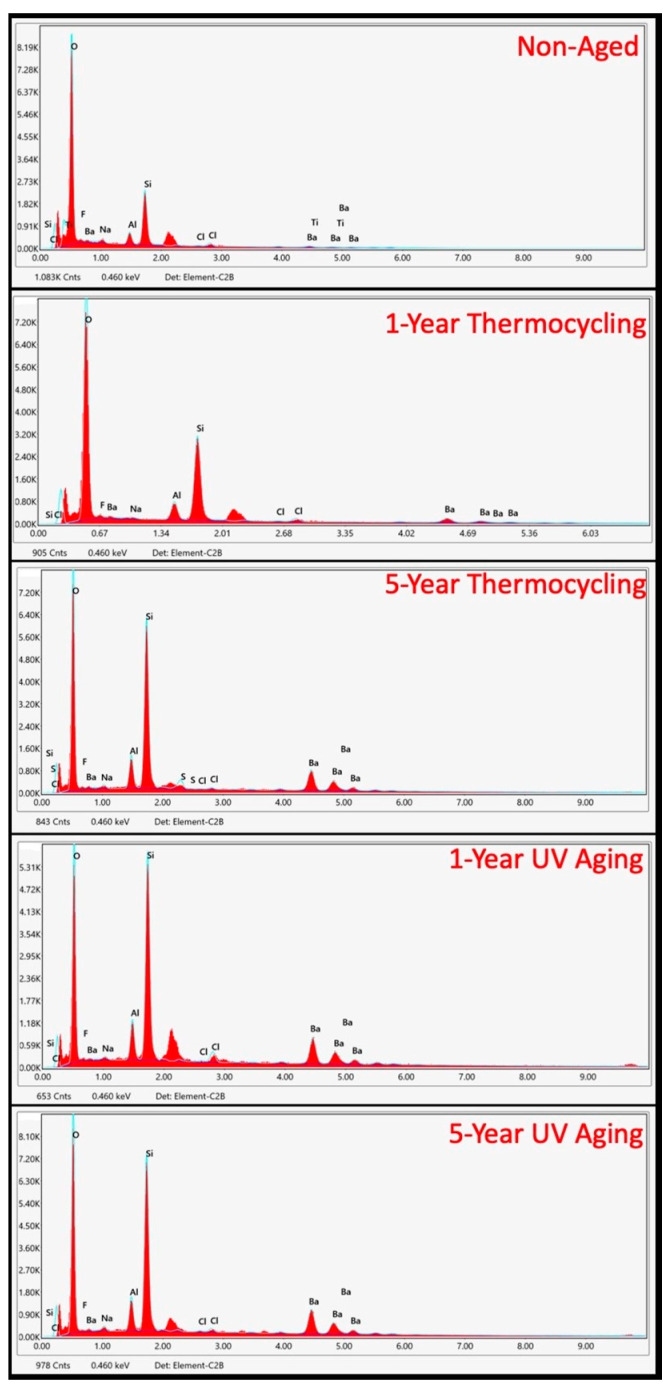
Chemical analysis of the surface of the CAD/CAM material after aging procedures.

The EDAX analysis identified the presence of carbon (C), oxygen (O), silicon (Si), barium (Ba), and aluminum (Al) elements across all groups. The results indicated that the wear occurred at the matrix level, which was evidenced by the increase in measurable inorganic filler ratios following the aging procedures. In the control group, the Si ratio measured by weight was determined to be 15.02%, while the Ba ratio was 3.83%. In contrast, these ratios increased significantly to 26.83% and 34.88%, respectively, in the samples subjected to five years of ultraviolet (UV) aging. In the samples subjected to a five-year thermocycling, the weight percentages of Si and Ba were constrained to 26.61% and 31.05%, respectively (Figure 4).

**Figure 5:**
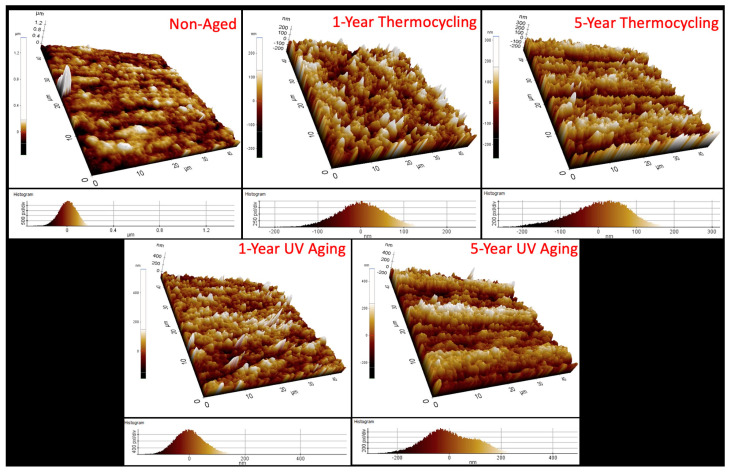
AFM images of the surface of the CAD/CAM material after aging procedures.

The images captured during the AFM analysis correspond closely with the SEM images, as well as the Ra and Rz measurements. Notably, the control group displayed the smoothest and most uniform microphotographs among all groups. In contrast, surfaces that underwent the aging procedure were found to exhibit irregular topography, characterized by cracks and pronounced protrusions. The observation of deep slits aligned with the trajectory of the wear pattern, as evidenced by SEM images following five years of UV aging, constitutes significant data (Figure 5).

Upon examining the microhardness data, a noteworthy finding emerged: the measurements demonstrated a significant decrease in response to the aging procedure, the cycle, and their interaction (*p* < 0.001, F = 217.3; *p* < 0.001, F = 211.2; *p* = 0.002, F = 11.0, respectively). The control group recorded the highest microhardness value at 75.63 VHN, while the samples subjected to 5 years of thermocycling and 1 year of UV aging exhibited statistically similar microhardness values (*p* > 0.05) (Table 2). Furthermore, Spearman’s rho correlation coefficient indicates a statistically significant negative correlation between the microhardness values of the samples and the Ra and Rz (*p* < 0.001; r = −0.918; *p* < 0.001; r = −0.899, respectively) (Table 3).

**Figure 6:**
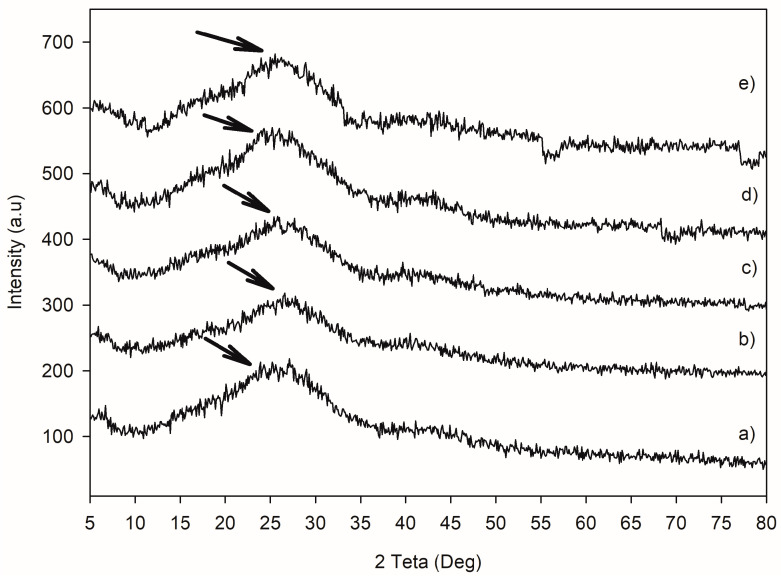
XRD analysis of the surface of the CAD/CAM material after aging procedures: (**a**) non-aged; (**b**) 1-year thermocycling; (**c**) 5-year thermocycling; (**d**) 1-year UV aging; (**e**) 5-year UV aging. Black arrows indicate the characteristic peaks of the surfaces.

Additionally, the XRD analysis showed that the samples exhibited a broad peak around 20°, with all samples displaying a similar amorphous phase (Figure 6). This suggests that the effects of aging are not sufficiently pronounced to induce a phase change.

The SBS values exhibited significant variations based on the preferred aging procedure and cycle (*p* < 0.001; F = 102.2; *p* < 0.001; F = 17.1, respectively). Notably, the highest SBS value was recorded in the control group, followed by samples subjected to 1-year thermocycling, 5-year thermocycling, 1-year UV aging, and, finally, 5-year UV aging (Table 2).

The failure types of the repaired samples are presented in Table 4. Adhesive failure was the most prevalent, occurring at a rate of 48.3%. Cohesive failure was predominantly observed in samples that underwent 5-year UV aging, with a rate of 75%. In contrast, the mixed failure type was observed infrequently.

## 4. Discussion

Although resin nanoceramic restorations have shown promising mechanical properties in vitro, their translation to clinical performance is somewhat limited. The effectiveness of these materials in a clinical context is closely linked to their chemical stability in the oral cavity [28]. To mitigate the risk of catastrophic failures during the use of restorations, it is imperative to thoroughly elucidate the mechanical and chemical degradation processes of the material through an appropriate aging methodology [29]. The findings from the current study indicate that the aging process and the duration of the applied cycle significantly influenced the microhardness, surface roughness, and repair bond strength of the samples (*p* < 0.001). Consequently, the study’s null hypothesis was rejected.

In the review of dental literature pertaining to the aging applications of materials, it is important to note that the procedures of soaking in water, exposure to acidic environments, thermocycling, and boiling in distilled water are frequently utilized [30]. All of these methodologies share a common characteristic: they replicate an accelerated aging process by generating a synergistic effect resulting from hydrolytic and thermal degradation [31]. While a consensus among researchers is lacking, certain studies indicate that various methods yield comparable effects on the mechanical properties of hybrid CAD/CAM blocks that incorporate resin [10,19]. Bessa et al. reported that varying water temperatures in the thermocycling method induced repetitive cycles of contraction and expansion stress in the material, thereby facilitating a more practical and effective aging process [32]. Given the gathered information, thermocycling was selected as one of the preferred methods in the present study. However, Oliveira et al. conducted a comparative analysis of the effects of water aging and ultraviolet (UV) aging on resin-based dental materials. Their findings indicated that the UV aging procedure led to intermittent water condensation. Consequently, they underscored that the refractive index of the components comprising the polymeric matrix of resin materials was altered, resulting in a more significant color change occurring in a substantially shorter duration when compared to water aging [33]. The current research team recognized the significance of elucidating the effects of UV aging, a contemporary approach that serves to inform the experimental protocols of researchers. Consequently, this aspect was regarded as essential and subsequently incorporated into the current study.

Surface roughness is a critical characteristic influencing the mechanical properties of polymer-based dental materials. It serves as a vital parameter in the assessment of surface properties [34]. Ardu et al. highlighted that surface roughness serves as a critical indicator of the aesthetic stability and durability of restorations [35]. In the current study, surface roughness measurements were conducted to elucidate the mechanical properties of CAD/CAM resin nanoceramic in relation to aging and repair bond strength. The results indicated a significant increase in both Ra and Rz parameters following the aging procedures (*p* < 0.001). Furthermore, a positive correlation was observed between the measured values and the increase in cycle time. Kim et al. reported that the aging process markedly increased the surface roughness of all materials evaluated in their study. This research focused on assessing the surface properties and mechanical strength of five distinct dental CAD/CAM restorative materials following exposure to thermocycling and mechanical loading. It was highlighted that the highest surface roughness values (Sa and Sq) among the materials tested were observed in the Cerasmart group. Furthermore, the researchers indicated the presence of localized surface uplift in the AFM images following an aging process, which corroborated the roughness data. Additionally, significant alterations in the microstructure of Cerasmart were noted in SEM images [34]. The findings presented herein align closely with our current research outcomes, both qualitatively and quantitatively. An additional in vitro study that examined the effects of various aging protocols on CAD/CAM resin nanoceramics indicated a significant increase in surface roughness compared to the control group, regardless of the alterations made to the aging procedure. Notably, the researchers reported that the Ra value recorded after a 3-month water soaking application, which was preferred as the aging procedure, measured 76.8 nm. This value increased to 88.1 nm after 9 months, highlighting a positive correlation between the Ra value and the duration of exposure. Furthermore, an important finding from the study reveals that the Ra value observed in samples subjected to a 1-year thermocycling application was limited to 69.8 nm, a figure that was statistically comparable to the surface roughness of the control group. This finding suggests that the effects observed will vary depending on the specific aging procedure employed [31]. The findings of the current study substantiate the researchers’ observations, revealing that the Ra value of 37.45 μm, measured after five years of thermocycling, is statistically comparable to the Ra value of 40.79 μm observed in samples subjected to one year of UV aging. This phenomenon may be attributed to the underlying mechanisms associated with aging processes. It is well-established that the temperature fluctuations experienced during thermal cycling induce thermal fatigue in restorative materials when exposed to humid conditions. Heat transfer occurs through convection, leading to the formation of thermal boundary layers and uneven temperature distributions. These variations can induce thermal stresses due to differing thermal expansion coefficients or non-uniform material properties. In multiphase materials such as composites, the expansion behaviors of filler particles can differ, resulting in additional internal stresses, particularly at the matrix–filler interface. Such processes contribute to the deterioration of the internal integrity of the materials. Cyclic temperature fluctuations primarily impact the surface layer, where stress gradients are most pronounced, thus heightening the risk of degradation. This leads to an enhancement in surface roughness [36]. In the UV aging procedure, alongside the thermal effects observed during thermocycling, there exists an interaction with unreacted amines present within the polymer matrix. These unreacted amines have the capacity to absorb UV light, subsequently leading to the formation of molecules that possess elevated energy states [3]. The observed increase in surface roughness during ultraviolet aging may primarily account for the more significant alterations noted. Existing literature indicates that aging processes typically result in an increase in surface roughness; however, these enhancements are generally deemed clinically acceptable, as they remain below the threshold of 0.2 μm, which is critical for in vivo bacterial adhesion [19,37]. In the present study, the Ra values measured following the aging process are significantly elevated beyond the established threshold. This variation in findings may be attributed to the differing aging procedures employed in the methodologies of the respective studies, as well as the fact that the cycle durations were restricted to 28 days and 3 months.

Microhardness serves as a relative indicator of a material’s resistance to external indentation forces and is recognized as a predictor of the strength of restorations in clinical applications [37]. In the present study, the inclusion of microhardness measurement in the methodology was deemed essential for predicting the clinical applicability of the restoration, detailing the effects of the aging procedures employed, and accurately correlating the results. A previous research assessed the mechanical properties of four distinct CAD/CAM resin nanoceramics, including Cerasmart, following various aging procedures. The findings indicated a significant reduction in microhardness across all experimental groups, with Cerasmart exhibiting the lowest microhardness values following each procedure when compared to the other materials evaluated. Among the findings reported, it is noteworthy that the microhardness value within the Cerasmart group decreased from 64.1 VHN to 59.0 VHN after merely 7 days of immersion in water. Additionally, following the thermocycling procedure, the microhardness further declined to 58.0 VHN. The researchers elucidated that the aging processes typically result in a downward trend in microhardness values. However, the variations in these decreasing values, contingent upon the specific aging procedure employed, may reflect the technical differences inherent in the respective methodologies [10]. In a similar vein, Kim et al. conducted a comprehensive study on the interaction of Cerasmart with water soaking, thermocycling, and mechanical loading procedures. Their results revealed that the lowest microhardness value was observed in the Cerasmart group following all aging procedures. The researchers noted a statistically significant decrease in the microhardness across the sample groups post-aging, which they considered to be a predictable outcome. They emphasized that the significant differences identified among the various aging procedures provided valuable insights for in vitro studies. Furthermore, the study highlighted an increase in the measured rates of the elements O, C, Si, and Ba following aging, which the researchers argued could be correlated with the observed loss of microhardness [34]. The findings of the current study align closely with both the observed reduction in microhardness following aging and the results obtained from the EDAX analysis. The decrease in microhardness identified in Cerasmart is likely attributable to water absorption. Given that all aging procedures employed in this investigation involve a humid environment, it is well established that water absorption leads to swelling within the resin matrix network, subsequently resulting in a reduction in frictional forces among the polymer chains [28]. The process initiates hydrolytic degradation of ester, urethane, amide, and siloxane bonds within the structure of resin nanoceramics. This degradation may lead not only to a reduction in hardness but also to the formation of additional voids on the surface [38]. This situation elucidates the observed increase in the concentrations of O, C, Si, and Ba elements on the surface, as well as the corresponding decrease in microhardness. Nevertheless, there are numerous studies in the literature that indicate that, despite the absorption of water by Cerasmart after aging, a reduction in microhardness was not detected. According to the researchers, the principal explanation for this observation is that the Bis-MEPP and DMA monomers present in Cerasmart mitigate the risk of cross-linking and monomer conversion, thereby enhancing resistance to hydrolytic degradation [28,39]. The divergence in findings may be attributed to the fact that the preferred aging procedure utilized in one study involved dry storage, whereas the thermocycling regimen employed in the other was comparatively short-term.

Restoration fractures may occur as a result of sudden trauma in the short term, or they may manifest after prolonged fatigue associated with masticatory functions over time [40]. The prevailing consensus in the literature indicates that repair should be regarded as a preferred alternative to restoration or replacement for suitable defects. This approach is not only cost-effective but also simplifies the overall process [41]. Upon reviewing the existing studies that assess the efficacy of repair procedures, it becomes evident that the literature predominantly emphasizes surface treatments designed to enhance repair bond strength. Furthermore, there is a paucity of research investigating the variations in repair success rates between aged and non-aged restorations [42]. The current researchers have not identified any literature that evaluates the repair bond strength in samples subjected to different aging methods. Addressing this gap, the present study assessed the repair bond strength in both aged samples, treated through various procedures, and unaged samples. Bessa et al. similarly investigated the impact of aging on the repair bond strength of CAD/CAM monolithic materials, centering their research on a comparable theme. Their findings indicated that the repair bond strengths of aged samples were consistently lower, independent of the surface cleaning method employed or the surface treatment administered. Notably, the SBS recorded in the analyzed aged samples was 7.70 MPa; this value was statistically comparable to the 6.62 MPa observed in the unaged control group. The researchers indicated that they observed alterations in the Weibull modulus and elevated characteristic strength values of the material following the application of thermocycling, which simulated a duration equivalent to one year of clinical use. They noted that the significant reduction in repair bond strength could be attributed to changes in the material’s properties resulting from aging. The study indicated that a mixed mode of failure was frequently identified through the fractographic analysis of the results obtained. This observation was interpreted as a reflection of the repair process in relation to the adhesive procedure, which does not preclude its clinical application [32]. In a recent investigation that included CAD/CAM resin nanoceramic material in the sample group, the repair bond strength of both non-aged and in situ aged samples was assessed. The findings indicated that aging led to a significant reduction in the SBS values, which was attributed to an increase in surface roughness resulting from the aging process and a corresponding rise in the concentration of inorganic fillers as revealed through an EDAX analysis. Furthermore, it was observed that the SBS values achieved through various surface treatments in unaged samples could be elevated to as much as 14.27 MPa; however, the values recorded following aging exhibited a dramatic decline, decreasing to as low as 0.17 MPa. Failure analysis demonstrated a mixed failure rate of 74% [43]. The results of the current study align with the established understanding that aging leads to a significant reduction in SBS values. As presented in Table 2, the repair bond strength values range from 11.54 MPa to 6.61 MPa. The observed variability in the data may be attributable to the classification of the samples as resin nanoceramics. However, these samples possess differing compositions in commercial production, which may result in varying effects of aging. Additionally, a notable distinction in this study is the predominance of adhesive failure as the most frequently observed mode. This phenomenon may be linked to the absence of various surface treatments in the methodology employed, which, subsequently, did not yield an enhancement in repair bond strength. It is widely acknowledged in the literature that adhesive failure is indicative of reduced SBS, while cohesive and mixed failures are suggestive of increased SBS [17].

Every in vitro study inherently possesses certain limitations. The most significant limitation of the current research is the reliance on a single resin nanoceramic, with the samples not being produced in an anatomically relevant form and without the use of a CAD/CAM system. Furthermore, as the primary objective of the current study is to investigate the fundamental effects of aging processes on materials in isolation, the replication of complex biomechanical stresses typical of the oral environment would considerably complicate the experimental design and impede the interpretation of the results. Consequently, masticatory forces were not simulated in this study. Although artificial aging methods were effectively employed to approximate intraoral conditions, the impacts of saliva—along with its constituent enzymes, ions, proteins, and even microbiota—were not taken into account. The influence of salivary enzymes and oral biofilms on the acceleration of resin nanoceramic degradation has yet to be thoroughly investigated and warrants focused research attention. Consequently, it is essential that the current results are contextualized with comparisons to both in vitro studies and clinical data pertaining to in situ aging.

## 5. Conclusions

In light of the constraints inherent to the current in vitro study, the following conclusions may be formulated:

1. The aging procedures and applied cycle time exert a significant influence on the surface properties and repair bond strength of CAD/CAM resin nanoceramics (*p* < 0.001).
2. An increase in surface roughness, accompanied by a decrease in microhardness and repair bond strength, was observed in aged CAD/CAM resin nanoceramic samples (*p* < 0.001).
3. Samples that underwent a 5-year UV aging process demonstrated the highest surface roughness, accompanied by the lowest values in microhardness and repair bond strength.

## Figures and Tables

**Table 1:** Materials utilized in the research.

Substance	Category	Components
Cerasmart	Resin nanoceramic CAD/CAM block	Bis-MEPP ^1^, UDMA ^2^, dimethacrylate, 71% silica (20 nm), barium glass (300 nm)
Ceramic Primer II	Silane	Silane, phosphate monomer, methacrylate, ethanol
G-Premio Bond	Universal adhesive	4-MET ^3^, phosphate monomer, thiophosphate monomer, dimethacrylate, acetone, water, photoinitiator
G-ænial Universal Flo	Flowable composite resin	Matrix (31% wt): UDMA ^2^, Bis-MEPP ^1^, TEGDMA ^4^ Filler (69% wt, 50% vol): Silicon dioxide (16 nm), Strontium glass (200 nm), Pigment Initiator: Photo initiator (trace)

¹ Bis-MEPP: bisphenol-A ethoxylate dimethacrylate. ² UDMA: urethane dimethacrylate. ³ 4-MET: 4-methacryloxyethyl trimellitic acid. ^4^ TEGDMA: triethyleneglycol dimethacrylate.

**Table 2:** Mean, median, and statistical comparison of Ra, Rz, microhardness, and SBS in aged and non-aged samples.

		5-Year UV Aging	1-Year UV Aging	5-Year Thermocycling	1-Year Thermocycling	Non-Aged (Control)	*p*
	Mean ± SD	63.13 ± 1.24 ^a^	68.12 ± 0.85 ^b^	68.18 ± 0.95 ^b^	71.31 ± 0.77 ^c^	75.63 ± 1.45 ^d^	<0.001 ^1^
	Median	63.45 (60.60–64.40)	68.30 (66.50–69.20)	68.35 (67.00–70.10)	71.05 (70.50–72.80)	75.35 (73.90–78.60)	
SBS	Mean ± SD	6.61 ± 0.67	8.64 ± 0.48	10.62 ± 2.04	11.32 ± 0.63	11.54 ± 0.74	<0.001 ^2^
	Median	6.40 (6.00–7.70) ^b^	8.65 (8.10–9.50) ^b^	9.75 (9.10–15.80) ^a^	11.35 (10.30–12.40) ^a^	11.75 (10.30–12.70) ^a^	
Ra	Mean ± SD	61.77 ± 0.24	40.79 ± 0.34	37.45 ± 0.34	36.08 ± 0.27	0.05 ± 0.02	<0.001 ^2^
	Median	61.79 (61.34–62.10) ^d^	40.84 (40.12–41.23) ^cd^	37.38 (37.00–38.00) ^bc^	36.00 (35.67–36.81) ^ab^	0.04 (0.02–0.10) ^a^	
Rz	Mean ± SD	271.57 ± 4.02 ^a^	213.90 ± 3.79 ^b^	200.61 ± 6.22 ^c^	187.91 ± 3.85 ^d^	0.22 ± 0.06 ^e^	<0.001 ^1^
	Median	271.26 (265.43–281.22)	213.62 (208.77–219.28)	200.03 (189.89–215.81)	188.47 (180.45–193.47)	0.22 (0.14–0.31)	

^1^ One-way analysis of variance test statistic, ^2^ Kruskal Wallis test statistics, ^a–e^: There is no difference between groups with the same letter.

**Table 3:** Correlation analysis results.

		Microhardness	SBS	Ra
SBS	r	0.796		
	*p*	<0.001		
Ra	r	−0.918	−0.849	
	*p*	<0.001	<0.001	
Rz	r	−0.899	−0.852	0.954
	*p*	<0.001	<0.001	<0.001

r: Spearman’s rho correlation coefficient.

**Table 4:** Distribution of failure modes.

Failure Mode	5-Year UV Aging	1-Year UV Aging	5-Year Thermocycling	1-Year Thermocycling	Non-Aged (Control)	Total	*p* *
Adhesive	2 (16.7%) ^a^	4 (33.3%) ^ab^	5 (41.7%) ^ab^	8 (66.7%) ^ab^	10 (83.3%) ^b^	29 (48.3%)	0.01
Cohesive	9 (75%) ^a^	7 (58.3%) ^ab^	5 (41.7%) ^ab^	1 (8.3%) ^b^	1 (8.3%) ^b^	23 (38.3%)	
Mix	1 (8.3%)	1 (8.3%)	2 (16.7%)	3 (25%)	1 (8.3%)	8 (13.3%)	

* Chi-square test, ^a,b^: There is no difference between groups with the same letter.

## Data Availability

The original contributions presented in the study are included in the article, further inquiries can be directed to the corresponding author.
